# Using a convolutional neural network for classification of squamous and non-squamous non-small cell lung cancer based on diagnostic histopathology HES images

**DOI:** 10.1038/s41598-021-03206-x

**Published:** 2021-12-13

**Authors:** Anne Laure Le Page, Elise Ballot, Caroline Truntzer, Valentin Derangère, Alis Ilie, David Rageot, Frederic Bibeau, Francois Ghiringhelli

**Affiliations:** 1 Department of Pathology, Caen University Hospital, Normandy University, Caen, France; 2 Platform of Transfer in Biological Oncology, Georges François Leclerc Cancer Center—UNICANCER, 1 rue du Professeur Marion, 21000 Dijon, France; 3 Genomic and Immunotherapy Medical Institute, Dijon University Hospital, 14 rue Paul Gaffarel, 21000 Dijon, France; 4 University of Burgundy-Franche Comté, Maison de l’université Esplanade Erasme, 21000 Dijon, France; 5 UMR INSERM 1231, 7 Boulevard Jeanne d’Arc, 21000 Dijon, France; 6 Department of Medical Oncology, Georges François Leclerc Cancer Center—UNICANCER, 1 rue du Professeur Marion, 21000 Dijon, France

**Keywords:** Lung cancer, Computational science, Diagnostic markers

## Abstract

Histological stratification in metastatic non-small cell lung cancer (NSCLC) is essential to properly guide therapy. Morphological evaluation remains the basis for subtyping and is completed by additional immunohistochemistry labelling to confirm the diagnosis, which delays molecular analysis and utilises precious sample. Therefore, we tested the capacity of convolutional neural networks (CNNs) to classify NSCLC based on pathologic HES diagnostic biopsies. The model was estimated with a learning cohort of 132 NSCLC patients and validated on an external validation cohort of 65 NSCLC patients. Based on image patches, a CNN using InceptionV3 architecture was trained and optimized to classify NSCLC between squamous and non-squamous subtypes. Accuracies of 0.99, 0.87, 0.85, 0.85 was reached in the training, validation and test sets and in the external validation cohort. At the patient level, the CNN model showed a capacity to predict the tumour histology with accuracy of 0.73 and 0.78 in the learning and external validation cohorts respectively. Selecting tumour area using virtual tissue micro-array improved prediction, with accuracy of 0.82 in the external validation cohort. This study underlines the capacity of CNN to predict NSCLC subtype with good accuracy and to be applied to small pathologic samples without annotation.

## Introduction

The standard of care in first line treatment of Non-Small Cell Lung Cancer (NSCLC) patients is based on chemoimmunotherapy or tyrosine kinase^1,2^. Treatment is assigned on the basis of specific histologic and genomic characteristics of the patient’s tumour^3^. In a first step, NSCLC must be classified into a particular histological type: non-squamous NSCLSC versus squamous cell carcinoma. This classification is essential for further molecular examination of the tissue sample to orient patients towards the optimal therapeutic treatment^4^. In case of non-squamous NSCLC, it is mandatory to obtain a list of molecular biomarkers, such as *EGFR* or BRAF V600E mutations, or *ALK* and *ROS1* rearrangements^5^. In addition, many emerging biomarkers require histological material for adenocarcinoma NSCLC (Met, NRG1, NTRK) but also for non-squamous NSCLC (PI3KCA, HRAS)^6^. However, while the molecular and histological material needed for treatment determination is increasing, the amount of histologic tumour tissue available is often small. Therefore, strategies that can help to reduce the material required for histological assessment will be helpful.

The development of Artificial Intelligence algorithms, which can be used to automatically classify histological slides, opens new perspectives in virtual and digital pathology. For example, in the setting of lung cancer, automatic analysis of whole-slide images of lung tumour resection has recently been studied to predict survival outcomes^7^, and can be used to predict histological type or mutational status^8^. However, such data are not relevant for clinical use because in most cases, pathologists only have a small tumour biopsy, or cytology fine needle aspiration.

To limit the volume of material required for histological diagnosis, we propose a deep learning convolutional network aimed at predicting the histological classification of non-squamous versus squamous cell carcinoma. Our analysis was based on tumour biopsy using whole tissue from biopsy or virtual TMA, based on annotation of the tumour zone.

## Results

### Population description

For the learning set, we included 132 HES slides from Dijon. These samples comprised 66 non-squamous and 66 squamous samples. Samples were obtained from primary lung tumour for all cases. The median tissue area was 11.734 [0.158–111.227] mm^2^ and the median tumour tissue area was 0.177 mm^2^ [0.002–1.088]. For the validation set, we included HES slides from Caen (n = 65; 45 non squamous and 20 squamous samples).

In the training and validation cohorts, no cytologic specimen was included, the sets were built with brushing and transbonchial small biopsies. In the test cohort, only one cytological was included (from pericarium liquid), the other specimens were either transbronchial or brushing small biopsies comparable to those included in the training and validation sets.

We also randomly selected 60 H&E slides from the LUAD and LUSC cohorts from the TCGA database, 30 for non-squamous patients and 30 for squamous patients.

###  A deep learning model for NSCLC subtype prediction using WSI classification

Our objective was to estimate a deep learning model to classify lung carcinoma subtypes using whole HES slides from tumour biopsy, regardless of the percentage of tumour cells contained in the biopsy. As described above, the learning cohort was decomposed into internal training, validation and test sets (Table 1).

**Table 1:** Description of learning and external validation cohorts.

	Non squamous cell carcinoma	Squamous cell carcinoma
**Learning cohort**		
Training sample (n = 78)	39 patients (77 640 tiles)	39 patients (62 496 tiles)
Validation sample (n = 26)	13 patients (23 750 tiles)	13 patients (21 801 tiles)
Test sample (n = 28)	14 patients (22 911 tiles)	14 patients (20 322 tiles)
**External validation cohort**		
External sample (n = 65)	45 patients (464 022 tiles)	20 patients (106 727 tiles)

**Figure 1:**
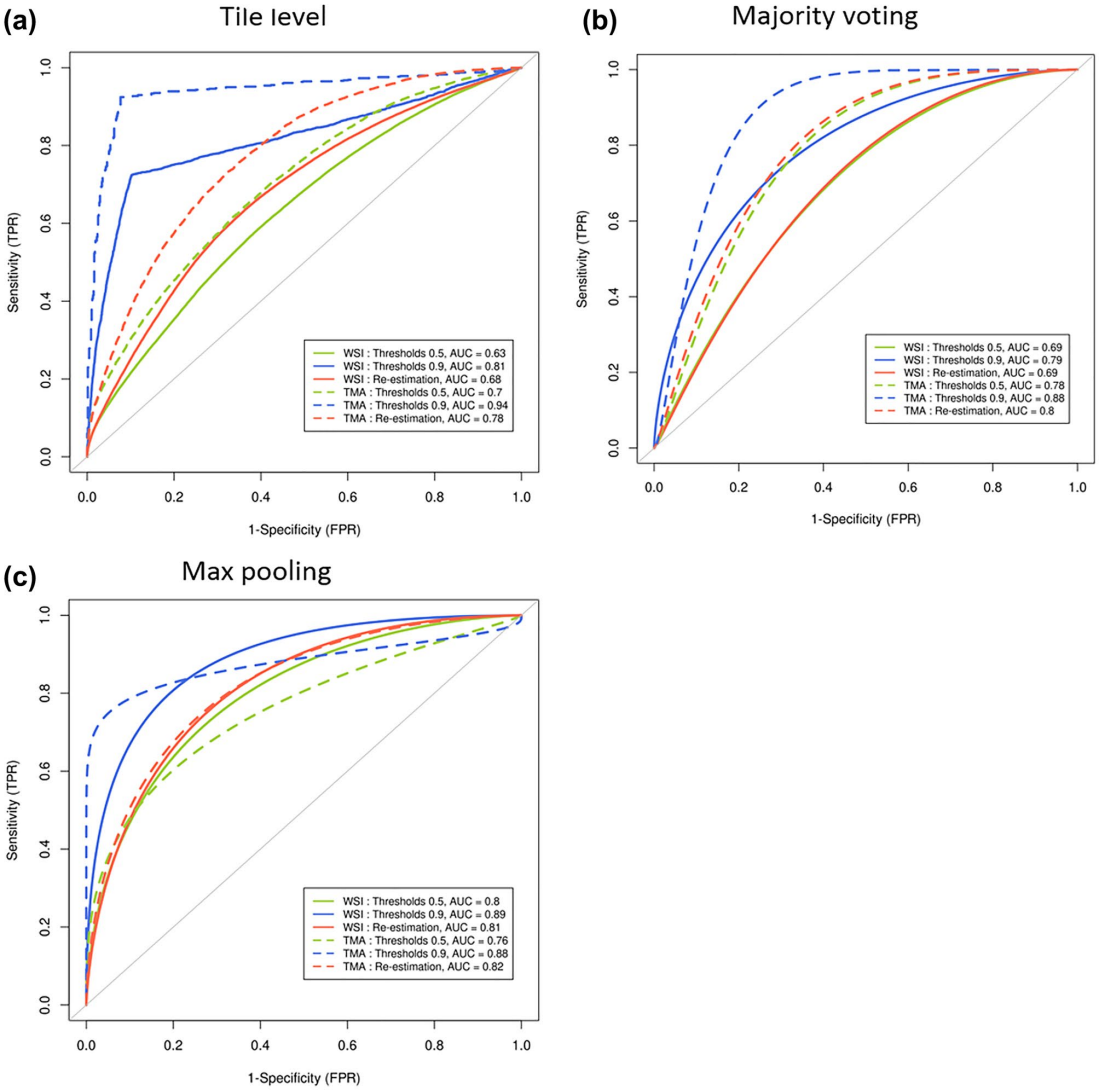
Evaluation of abilities of the different strategies to predict the class of tiles in the external validation cohort. ROC curves at tile level (**a**), patient level according to majority voting (**b**) and max pooling (**c**). Green, blue and red lines correspond respectively to threshold equal to 0.5, threshold equal to 0.9 and re-estimation using filter kernel. Solid and dashed lines respectively correspond to the prediction of tiles from whole slides and TMA.

Using Inception V3 deep learning architecture, our CNN model was optimized using different approaches. First, we added a threshold for predictions to retain only tiles with high prediction level; in fact, it is expected that WSI include a large number of tiles without tumour cells, which would alter predictions with noisy information. A second strategy used a kernel filter to take into account the spatial environment of the tiles. At the tile level, accuracies from the resulting models underline that the threshold methodology is the best strategy, with values of 0.99, 0.87 and 0.85 respectively in the training, validation and test datasets. Similarly, our model had an accuracy of 0.85 (Table 2) and AUC of 0.81 (Fig. 1a) in the external validation cohort which underlines the robustness of the model and the absence of overfitting. Supplemental Fig. S1 shows the accuracy and loss across epochs for model estimation. An accuracy of 0.75 and an AUC of 0.78 were reached in the TCGA dataset.

**Table 2:** Accuracy achieved by the different strategies at tile level.

	Training	Validation		Test		External validation		TCGA	
	Overall	Overall	By class	Overall	By class	Overall	By class	Overall	By class
**WSI**									
*Thresholds: *NS = 0.5, S = 0.5	0.75	0.64	NS: 0.70 S: 0.57	0.58	NS: 0.66 S: 0.49	0.68	NS: 0.73 S: 0.44	0.53	NS: 0.62 S: 0.44
NS = 0.9, S = 0.9	0.99	0.87	NS: 0.88 S: 0.87	0.85	NS: 0.84 S: 0.86	0.85	NS: 0.89 S: 0.72	0.75	NS: 0.77 S: 0.73
*Re-estimation using filter kernel*	0.84	0.71	NS: 0.79 S: 0.62	0.63	NS: 0.71 S: 0.53	0.71	NS: 0.77 S: 0.48	0.53	NS: 0.69 S: 0.38
**TMA**									
*Thresholds*: NS = 0.5, S = 0.5	0.78	0.69	NS: 0.74 S: 0.63	0.65	NS: 0.68 S: 0.62	0.66	NS: 0.77 S: 0.49	0.57	NS: 0.59 S: 0.55
NS = 0.9, S = 0.9	0.99	0.83	NS: 0.84 S: 0.82	0.88	NS: 0.77 S: 0.92	0.92	NS: 0.92 S: 0.94	0.83	NS: 0.55 S: 0.94
*Re-estimation using filter kernel*	0.88	0.79	NS: 0.83 S: 0.76	0.73	NS: 0.75 S: 0.71	0.71	NS: 0.81 S: 0.56	0.57	NS: 0.66 S: 0.50

To classify the tumour slide, we pooled tile information using either max pooling or majority voting strategies. The best strategy was majority voting; using this strategy, our model had an accuracy of 0.73, 0.78 and 0.64 respectively in the learning, external validation and TCGA cohorts (Table 3). In the external validation cohort, the model had an AUC of 0.79 using majority voting (Fig. 1b) and 0.89 using max pooling (Fig. 1c). In the TCGA cohort, the model had an AUC of 0.67 using majority voting and 0.76 using max pooling.

**Table 3:** Accuracy achieved by the different strategies at patient level.

	Max pooling						Majority voting					
	Test		External validation		TCGA		Test		External validation		TCGA	
	Overall	By class	Overall	By class	Overall	By class	Overall	By class	Overall	By class	Overall	By class
**WSI**												
*Thresholds*: NS = 0.5, S = 0.5	0.71	NS: 0.71 S: 0.71	0.74	NS: 0.69 S: 0.85	0.64	NS: 0.47 S: 0.81	0.68	NS: 0.79 S: 0.57	0.71	NS: 0.80 S: 0.50	0.54	NS: 0.73 S: 0.35
NS = 0.9, S = 0.9	0.69	NS: 0.69 S: 0.69	0.74	NS: 0.69 S: 0.85	0.64	NS: 0.47 S: 0.81	0.73	NS: 0.77 S: 0.69	0.78	NS: 0.76 S: 0.85	0.64	NS: 0.57 S: 0.71
*Re-estimation using filter kernel*	0.61	NS: 0.57 S: 0.64	0.81	NS: 0.77 S: 0.90	0.54	NS: 0.73 S: 0.32	0.71	NS: 0.79 S: 0.64	0.73	NS: 0.80 S: 0.60	0.52	NS: 0.77 S: 0.29
**TMA**												
*Thresholds*: NS = 0.5, S = 0.5	0.79	NS: 0.79 S: 0.79	0.79	NS: 0.79 S: 0.80	0.72	NS: 0.57 S: 0.87	0.82	NS: 0.93 S: 0.71	0.73	NS: 0.83 S: 0.50	0.58	NS: 0.7 S: 0.47
NS = 0.9, S = 0.9	0.68	NS: 0.67 S: 0.70	0.80	NS: 0.79 S: 0.83	0.73	NS: 0.52 S: 0.92	0.68	NS: 0.67 S: 0.70	0.82	NS: 0.81 S: 0.83	0.73	NS: 0.52 S: 0.92
*Re-estimation using filter kernel*	0.79	NS: 0.79 S: 0.79	0.77	NS: 0.79 S: 0.75	0.67	NS: 0.83 S: 0.50	0.82	NS: 0.86 S: 0.79	0.73	NS: 0.79 S: 0.60	0.57	NS: 0.77 S: 0.37

### Prediction using virtual TMA analysis of WSI

In order to improve classification and computational time, we created a virtual TMA, using a circle with a radius of 500 µm from the centroid of the annotation drawn by the pathologist. The computational time for predictions on TMA was 18 times less than on the entire slide. This strategy also has a benefit that can translate in clinical routine with quick annotation by a pathologist, who just clicks on the tumour core instead of contouring the whole tumour. When we applied the unique model trained in WSI on TMA, the accuracy findings confirmed that using this gating strategy, the threshold methodology was also the best strategy, with model accuracy of 0.99, 0.83 and 0.88 in the training, validation and test datasets at the tile level. Similarly, the model had accuracy of 0.92 (Table 2) and an AUC of 0.94 (Fig. 1a) in the external validation cohort. An accuracy of 0.83 and an AUC of 0.77 were reached in the TCGA cohort.

We then used the TMA strategy to predict tumour slide classification. Using majority voting, the model had an accuracy of 0.68, 0.82 and 0.73 respectively in the learning, external validation and TCGA cohorts (Table 3). In the external validation cohort, the AUC was equal to 0.88 for the both strategies max pooling and majority voting (Fig. 1b,c).

In the TCGA dataset, the AUC was equal to 0.63 using majority voting and 0.79 using max pooling.

**Figure 2:**
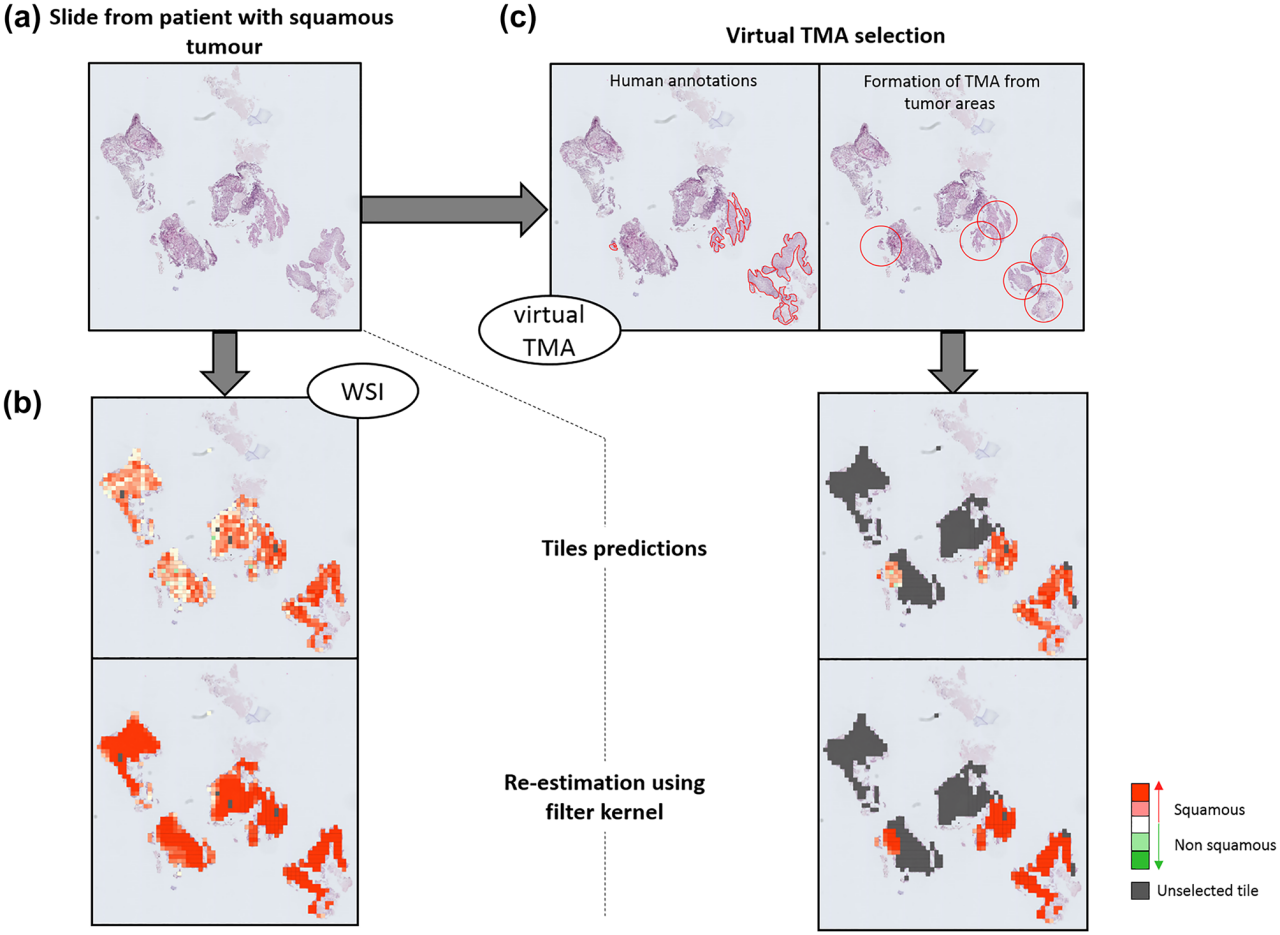
Prediction procedure for a squamous tumour biopsy section. (**a**) Representation of original slide. (**b**,**c**) Steps for prediction analysis based on (b) Whole Slide Image (WSI) or (**c**) virtual TMA strategy. Heatmaps show for each tile probability of being predicted as squamous tumour (red) or non-squamous tumour (green); the highest probability was kept for coloration; grey was used for tiles non selected by the pathologist or removed by denoising step (tile containing more than 2/3 of white background).

**Figure 3:**
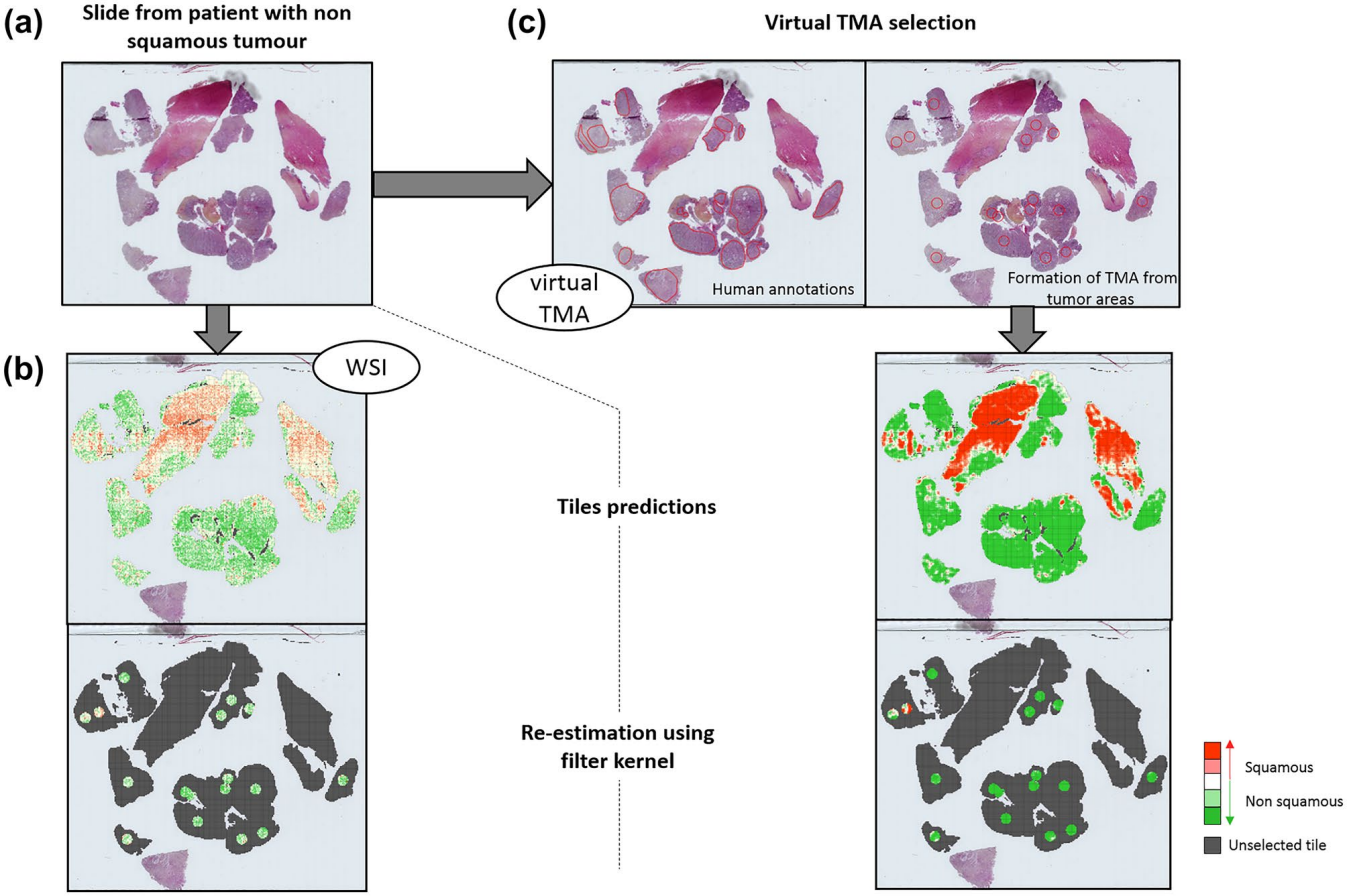
Predictions of tumour biopsy section containing non-squamous tumour. (**a**) Representation of original slide. (**b**,**c**) Steps for prediction analysis based on (**b**) whole Slide Image (WSI) or (**c**) virtual TMA strategy. Heatmaps show for each tile probability of being predicted as squamous tumour (red) or non-squamous tumour (green); the highest probability was kept for coloration; grey was used for tiles non selected by the pathologist or removed by denoising step (tile containing more than 2/3 of white background).

Figures 2 and 3 show two cases of tumour biopsy sections containing respectively squamous and non-squamous tumour, with the correct diagnosis and the predicted WSI diagnosis based on TMA or WSI analysis for each prediction step.

## Discussion

The diagnosis of NSCLC is based on morphological evaluation of tissue specimens. This analysis is the first step before addressing samples for molecular testing and therapy stratification^4^. One issue in the management of metastatic lung cancer is that in most cases, samples are cytological exams or small biopsies. Preservation of the sample in this clinical context for further molecular testing is important. Consequently, even if applying artificial intelligence on such a routine exam may seem irrelevant an experienced pathologist who is well trained in analysis of IHC staining like TTF1 and p40, it clearly assure a sparingly use of biopsy specimen. Our study, like previous reports, showed that the combination of digital pathology and machine learning has the potential to support this decision process in an objective manner^9^. In previous works, the application of deep learning to classify lung histological specimens yielded promising results in lung cancer^10–12^. However most of these reports only fostered on surgical samples.

In this study, we analysed whether a CNN-model (InceptionV3 CNN) could be used to differentiate squamous from non-squamous NSCLC, based on the initial tumour biopsy. This study was performed without taking into account the tissue type of the biopsy, or whether the sample was a cytological or histological sample. In this work, we addressed some technical points and show that the whole slide can be used to predict the histological subtype with good accuracy, without prior tumour tissue selection by the pathologist. Surprisingly, adding spatial information using kernel filter did not improve the classification. In contrast, adding quality check with a threshold to select only predictions with a good level of confidence improved the accuracy of the classification. These findings are not unexpected, since WSI include many non-tumour zones.

To improve the prediction, we also used a virtual TMA strategy. Based on the pathologist’s hand-drawn tumour annotations, TMA were created by tracing a circle with a radius of 500 µm from the centroid of this annotation. This strategy could easily be reproduced by a pathologist, who could click on the virtual slide to localize the tumour and obtain the prediction for the whole slide using only TMA restricted information.

We chose to estimate our model on whole-slide images because we believe stroma or connective tissues can also be within the tumor, i.e. tumor is made of tumor cells for sure, but also of stroma, immune and connective tissue. But it is also sure that predicting squamous or non-squamous subtype on restrictive connective tissue made no sense. We think that can explain why virtual TMA improve results. Khosravi et al.^13^ also observed an improvement when using TMA strategy.

The limitations of our study include the small sample size, and the small number of extracted image patches in some cases, which may limit the accuracy of the model. Moreover, epithelial lung tumours may be morphologically very different. In particular, the current World Health Organization classification is more complex and separates adenocarcinoma into several different subtypes, such as lepidic, solid, acinar, and papillary. Because of the small learning set, we did not include this information in the model, but using a larger learning set with further non-squamous subtype labelling would undoubtedly improve the capacity of the CNN model to predict histological types with greater accuracy. Further studies are warranted on this point. While the learning set was performed on lung biopsy, the model is validated on either cytological or pathological samples, and also on either lung biopsy or metastatic samples. This heterogeneity in the samples may induce some bias, and may limit the accuracy of the model. However, we chose this heterogeneity to better reflect the clinical reality of lung cancer diagnosis.

We compared our results to those obtained in lung cancer in other recent works^8,13–16^. In these works, models were trained on H&E TCGA slides, thus on hundreds of image slides. There were then evaluated on other public H&E slides. Coudray et al.^8^ get the best AUC, with a value of 0.97. Using machine learning models, Yu et al.^14^ get an AUC of 0.75. Other authors obtained AUC between these 2 values. Predictive abilities of our model are in the same range, although estimated on HES slides. We would like to remind that our objective was to propose a model that can be applied by pathologists belonging to French network using HE & Safran on HES diagnostic slides.

In summary, we trained and optimized an Inception V3 CNN model to classify the two common NSCLC subtypes using routine biopsy or cytological samples. Moreover, we established a virtual TMA strategy to improve predictions. Our results highlight the potential and limitations of CNN image classification models for morphology-based tumour classification.

## Methods

### Study population

The learning cohort comprised 132 NSCLC tumour biopsies (66 non squamous and 66 squamous samples) collected between 2015 and 2018 in the Department of Pathology of the Georges François Leclerc Cancer Center in Dijon, France.

The external validation cohort comprised 65 biopsy samples (45 non squamous and 20 squamous samples) from the University Hospital of Caen, France, using tumours collected between 2017 and 2019.

Whole slide histopathology images from 30 non-squamous and 30 squamous patients were taken from the LUAD and LUSC cohorts of the Cancer Genome Atlas (TCGA). Data were obtained from the National Cancer Institute Genomic Data Commons^17^.

Only patients from whom informed consent was obtained were included in this retrospective study. The present study was approved by the CNIL (French national commission for data privacy) and the Georges François Leclerc Cancer Center (Dijon, France) local ethics committee, and was performed in accordance with the Helsinki Declaration and European legislation.

### Pathological diagnosis

The pathological diagnosis (adenocarcinoma versus squamous cell carcinoma) was validated for all samples by a pathologist (ALLP). Pathological classification was performed using analysis of morphology on HES stained slides and TTF1 and p40 immunohistological analysis.

### Image processing

Formalin-fixed paraffin-embedded HES stained slides were digitised with a Nanozoomer HT2.0 (Hamamatsu) at 20× magnification to generate a whole slide imaging (WSI) file in ndpi format. We partitioned the WSI into non-overlapping 220 × 220 pixel tiles at 0.5 mm/pixel resolution (equivalent to 20× magnification) using QuPath v.0.2.3^18^.

In addition, tumour regions of each slide were manually annotated by a pathologist (ALLP). Then, the centroids of each annotation were calculated. A TMA was created based on a circle with a radius of 500 µm from the centre of the centroid of the annotation. The same tiling as described above was kept.

### Tile Pre-processing

Tiles were removed if they contained more than 2/3 of white background. The color channel values were normalized by Reinhard normalization to neutralize color differences between slides^19^. This normalization uses a linear transformation to match the mean and standard deviation between slides. The color channel values were scaled to a floating value range of [0, 1].

Training, validation and test sets were generated using respectively 60%, 20% and 20% of tiles. Tiles associated with a given slide were not separated, but associated with one of these sets to prevent overlap of slides between the three sets.

### Deep learning model

We estimated a model based on InceptionV3^20^. The idea behind the Inception architecture is to use a series of convolutional blocks to both decrease the number of parameters in the network and improve its performance. The main components of a convolutional block are convolutional and pooling layers. To make the algorithm more robust against image variations, and to add a regularisation effect, we applied data augmentation techniques. This included techniques such as randomly flipping the images left–right and up-down with additional random rotations.

The model was fully trained for one hundred epochs on the augmented training set, starting with an initial learning rate of 0.001, decaying by a factor of 0.9 every five epochs and using the Adam optimisation algorithm^21^ with a momentum of 0.9 and epsilon of 1e−7. We used a batch size of 100 tiles.

Due to an unequal number of extracted tiles for each class (unbalanced dataset), we used a weighted loss function allowing direct penalization of false predictions during the training process. Negative and false positives were equally penalized with a 1.5 factor.

### Patient inference

We then classified each tile and filtered out low-confidence predictions by using thresholding. Thresholds were determined by a grid search over each class, optimizing the correct classification rate^22^.

The CNN can be used directly as a classifier, but it predicts each tile independently and ignores spatial correlations. To take advantage of the neighbourhood of each tile, filter kernel algorithms aimed at extracting spatial information were used; the filter kernel takes advantage of the label distribution of neighbouring patches to re-estimate the output of CNNs. A logistic regression algorithm was used as the strategy for parameter estimation of the filter kernel^23^.

If the label of a tile is the same as the label of the neighbouring tiles, its probability will be increased. Conversely, it will have a lower probability when its label differs from that of its neighbours.

To classify the whole slide, we used two methods^24^. The first, called “majority vote”, assigned the most frequent class to the slide. The second, called “max pooling”, assigned the class with the highest probability to the slide.

These different strategies were applied on tiles from the whole slide as well as on tiles from TMA only in order to focus the results on tumour regions. More precisely, the training was the same in both strategies. A unique model was estimated based on tiles taken from whole slides of training set. This model was then evaluated on testing and external validation sets performing prediction either using tiles from whole slides, or using tiles restricted to TMA regions.

Receiver operating characteristic (ROC) curve and area under the curve (AUC) analysis were performed to evaluate the abilities of the different strategies to predict the class of tiles from whole slide and from TMA at slide and patient levels in the external validation cohort.

### Software

The deep learning model was implemented and trained using TensorFlow 2.1.0 and python 3.5. Calculations were performed using HPC resources from DNUM CCUB (Centre de Calcul de l’Université de Bourgogne).

Data analysis was performed using R statistical software (http://www.R-project.org/).

## Supplementary Information

Supplementary Figure S1.

## Data Availability

Images from training and validation cohorts, as well as the code used for statistical analysis are available from the corresponding author on reasonable request.
