# Supplementary material for: Using a convolutional neural network for classification of squamous and non-squamous non-small cell lung cancer based on diagnostic histopathology HES images
Source: Sci Rep. 2021 Dec 13;11:23912. doi: 10.1038/s41598-021-03206-x (PMC8669012; doi:10.1038/s41598-021-03206-x)
Supplement: Supplementary file 1 — Supplementary Figure S1. [file 41598_2021_3206_MOESM1_ESM.pptx]

## Slide 1
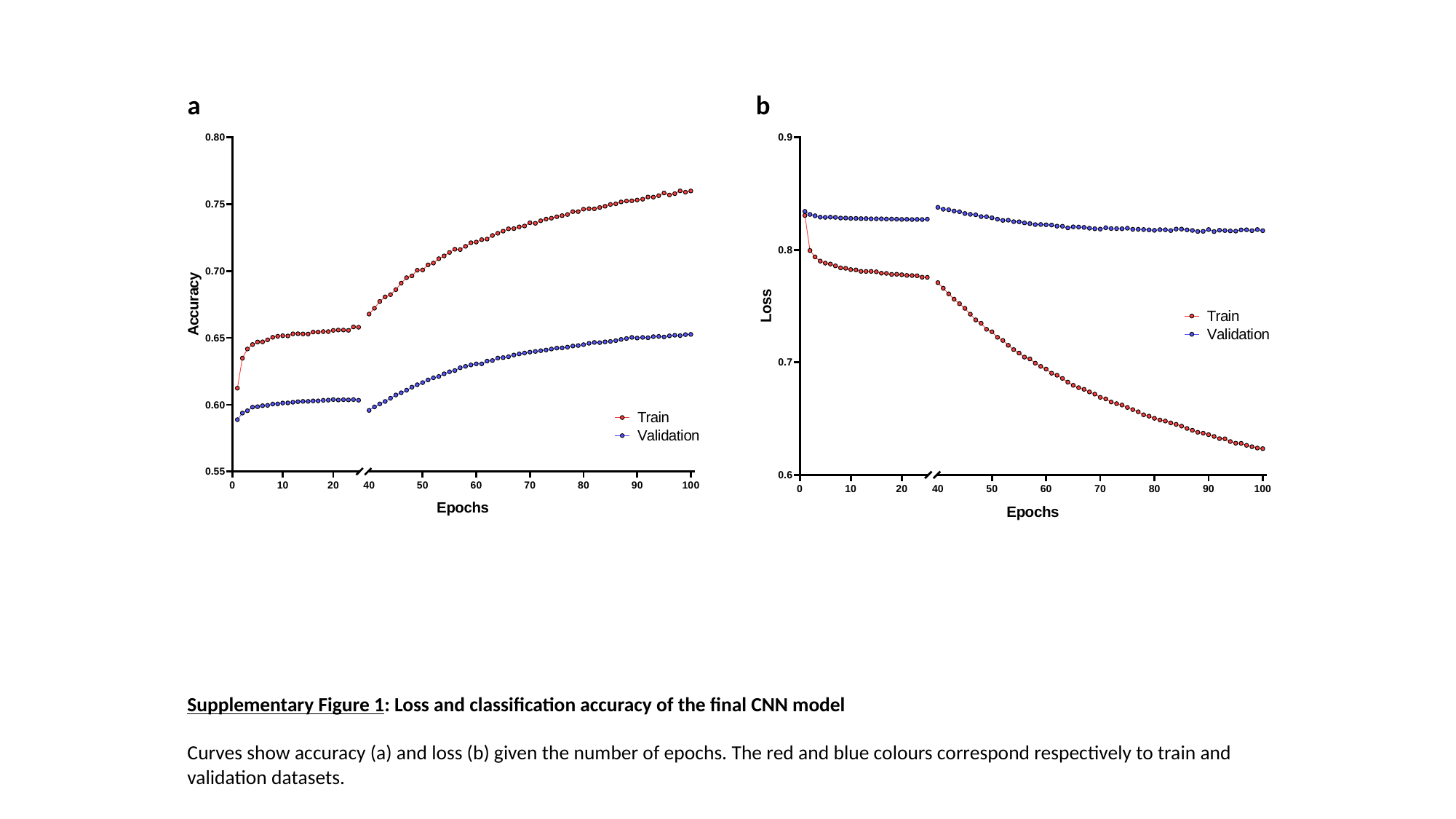

b
a
Supplementary Figure 1: Loss and classification accuracy of the final CNN model
Curves show accuracy (a) and loss (b) given the number of epochs. The red and blue colours correspond respectively to train and validation datasets.
